# Sarcopenia Index trajectories predict long-term mortality in super-elderly patients with sepsis: a retrospective cohort study

**DOI:** 10.3389/fmed.2026.1782726

**Published:** 2026-03-16

**Authors:** Jieyu Chen, Min Ma, Xiaoling Zhou, Binbin Chang

**Affiliations:** Department of Geriatrics, The General Hospital of Western Theater Command, Chengdu, China

**Keywords:** Group-Based Trajectory Modeling (GBTM), risk stratification, Sarcopenia Index (SI), sepsis, super-elderly

## Abstract

**Background:**

The dynamic trajectory of muscle mass during sepsis may hold superior prognostic value over static assessments, particularly in vulnerable super-elderly patients. This study aimed to identify distinct dynamic trajectories of the Sarcopenia Index (SI) using Group-Based Trajectory Modeling (GBTM) and investigate their association with 180-day mortality.

**Methods:**

This retrospective cohort study enrolled 210 super-elderly patients (aged >85 years) with sepsis. GBTM was employed to delineate SI trajectories over 60 days. The primary outcome was 180-day mortality. Kaplan–Meier analysis and multivariable Cox proportional hazards regression were used to assess the association between SI trajectories and mortality. The incremental predictive value of trajectory data was evaluated using C-index, Net Reclassification Improvement (NRI), and Integrated Discrimination Improvement (IDI).

**Results:**

Two distinct SI trajectories were identified: a “High-Level Group” (*n* = 88) and a “Low-Level Group” (*n* = 122). The Low-Level Group was characterized by lower baseline SI, poorer functional status, and higher prevalence of long-term bedridden status. The 180-day mortality rate was higher in the Low-Level Group (62.3% vs. 48.9%); however, this difference did not reach statistical significance in the unadjusted analysis (*p* = 0.072). After multivariable adjustment, assignment to the Low-Level trajectory remained an independent predictor of mortality (Adjusted HR = 1.64, 95% CI: 1.08–2.48, *p* = 0.020). Adding the SI trajectory to a clinical risk model led to a small but statistically significant improvement in risk reclassification (NRI = 0.020, *p* < 0.05), while discrimination gains were modest (IDI = 0.150, *p* = 0.078).

**Conclusion:**

A low and declining SI trajectory is an independent predictor of long-term mortality in super-elderly sepsis patients. Dynamic monitoring of SI provides incremental prognostic value over static assessments, offering a novel tool for early risk stratification and targeted interventions.

## 1 Introduction

Sepsis, a life-threatening organ dysfunction resulting from a dysregulated host response to infection, represents a major challenge in critical care medicine (1, 2). The annual epidemiological statistics indicate that it ranks among the most common sources of infection-related deaths worldwide, with an incidence of nearly 49 million and mortality rates of more than 20 percent (3). Sepsis especially affects the elderly who are bearing a disproportionate burden including more than two-thirds of the patient population in such areas as China (4). Han et al. (4) highlighted that protein-energy malnutrition and micronutrient deficiencies are prevalent in this demographic, which not only compromise immune function but also accelerate skeletal muscle degradation. This preexisting nutritional vulnerability, combined with immunosuppressive aging-associated risk, is especially high among the super-elderly (over 85 years old), in which host defense is compromised both synergistically by age-associated immunosenescence and persistent low-grade inflammation, resulting in a growing sepsis incidence and mortality with an almost 50 percent rate of occurrence (5).

At the same time, sarcopenia, which is a syndrome of progressive and generalized muscle mass, strength, and functional depletion, is also becoming more commonly discussed as the independent prognosis of critically ill patients (6, 7). It is approximated that nearly half of the patients in intensive care unit (ICU) develop sarcopenia or malnutrition, which causes a significant reduction in immune response, delayed recovery, prolonged duration of mechanical ventilation and hospital stay, which in turn increases the risk of mortality (8).

According to Kashani et al. (9), the Sarcopenia Index (SI) is a convenient, non-invasive technique of estimating muscle mass. The SI corrects the confounding effect of renal dysfunction by dividing the ratio of serum creatinine (a muscle metabolism product) and cystatin C (a renal functioning marker) and offers a specific indication of somatic muscular stores (10). This will especially be beneficial to super-elderly bedridden patients where the more conventional functional measures such as grip, strength or gait speed are often not feasible (11). Nonetheless, one, fixed, standardized SI measurement at admission cannot capture the variable and increasingly acute amortisation of muscle mass, which is driven by the systemic inflammatory response and the ubiquitin-proteasome pathway characteristic of the hypercatabolic state in sepsis (12). This acute muscle atrophy represents a critical knowledge gap, as the trajectory of muscle depletion, rather than merely its initial level, may hold superior prognostic value (13).

Group-Based Trajectory Modeling (GBTM) is a complex statistical model that allows the detection of latent subpopulations who have different longitudinal patterns that can be used as a new way of perceiving prognostic heterogeneity (14). It has not been used in a systematic study to follow dynamic changes in SI of super-elderly sepsis patients. Therefore, this study aimed to: (1) apply GBTM to outline unique dynamic pathways of the Sarcopenia Index after the onset of sepsis; (2) determine whether there are specific patterns in dynamic trajectories that are linked to 180-day mortality; and (3) evaluate whether dynamic trajectory data can help predict outcomes in the most vulnerable group by adding to the existing background of knowledge.

## 2 Methods

### 2.1 Study design and ethical considerations

This retrospective observational study was conducted at the Department of Geriatrics, the General Hospital of Western Theater Command, covering the period from January 2018 to December 2023. The study protocol was strictly aligned with the ethical principles outlined in the Declaration of Helsinki and received formal approval from the Ethics Committee of the General Hospital of Western Theater Command (Approval No. 2025EC3-ky014). Given the retrospective nature of the analysis, which utilized anonymized clinical data collected during routine care, the requirement for individual written informed consent was waived by the Institutional Review Board. All patient data were de-identified prior to analysis to ensure confidentiality.

### 2.2 Study population and eligibility criteria

The study cohort comprised super-elderly patients (aged > 85 years) who were hospitalized with a diagnosis of sepsis. Sepsis was defined according to the Sepsis-3.0 consensus criteria, necessitating a confirmed infection combined with an acute change in the Sequential Organ Failure Assessment (SOFA) score of ≥ 2 points.

To ensure the homogeneity of the study population and the reliability of the Sarcopenia Index (SI) calculations, strict exclusion criteria were applied: (1) length of hospital stay less than 24 h; (2) patients aged under 85 years; (3) pre-existing acute or chronic renal insufficiency requiring renal replacement therapy, which would fundamentally confound creatinine and cystatin C levels; and (4) insufficient longitudinal data for trajectory modeling, defined as fewer than three valid SI measurements during the follow-up period. Based on clinical outcomes at 180 days post-onset, participants were stratified into survival (*n* = 91) and non-survival (*n* = 119) groups for comparative analysis.

### 2.3 Data collection and definition of Sarcopenia Index

Comprehensive clinical data were retrospectively extracted from the electronic medical record system. Baseline demographic information, including age, gender, and history of long-term bedridden status (defined as immobilization for >14 days), was recorded. Vital signs at sepsis onset—specifically blood pressure, heart rate, respiratory rate, body temperature, and oxygen saturation—were documented. Comorbidities were systematically categorized into chronic conditions [e.g., hypertension, coronary heart disease, diabetes mellitus, COPD, history of stroke, chronic heart failure (NYHA class ≥ III), cognitive impairment] and acute events (e.g., new-onset cerebral infarction, intracranial hematoma, gastrointestinal bleeding, acute myocardial infarction, pulmonary embolism). Additionally, active malignancy, postoperative status, and specific sepsis etiologies (aspiration or COVID-19) were noted.

Laboratory parameters measured at onset included white blood cell count, platelet count, hemoglobin, high-sensitivity C-reactive protein (hs-CRP), procalcitonin (PCT), D-dimer, and albumin. Clinical severity was stratified using the APACHE II, SOFA, and Barthel Index scores assessed within 24 h of admission.

The Sarcopenia Index (SI) was calculated using the validated formula: SI = (Serum Creatinine [mg/dL]/Cystatin C [mg/L]) × 100. To capture longitudinal dynamic changes, SI measurements were obtained at five specific time points: within 24 h of sepsis onset (baseline), and subsequently at days 7, 15, 30 (1 month), and 60 (2 months) post-onset.

### 2.4 Group-Based Trajectory Modeling (GBTM)

Group-Based Trajectory Modeling (GBTM) was used in order to detect latent sub-populations with similar longitudinal patterns of muscle mass evolution. This semi-parametric method is capable of identifying different clusters in the population without any *a priori* assumptions regarding the trajectories that they follow. In models that had different groups (one to three group), censored normal curves were utilized to fit the model.

The combination of clinical interpretability and statistical criteria was used to select the model. Bayesian Information Criterion (BIC) was employed as a primary index of model fit with lower values representing better fit. Model adequacy was further evaluated using the average posterior probability (AvePP) of assignment, with a value > 0.7 for each group considered acceptable. The cubic polynomials were adopted to model the trajectories in order to be flexible to changes that are non-linear with time. The patients were allocated to the group of trajectory where they were most likely to belong.

### 2.5 Statistical analysis

The continuous variables were expressed by means + standard deviation (SD) in normally distributed cases or median [interquartile range, IQR] in the non-normally distribution cases. Categorical variables were represented in terms of numbers and percentages (*n*, %). Student *t*-test, Mann Whitney *U* test or Chi-square test, depending on the appropriateness, were used to perform intergroup comparison between trajectory classes.

The estimates of the survival were done through the Kaplan–Meier method and contrasted with the Log-rank test. Univariable and multivariate Cox proportional hazards regression models were built in order to determine independent predictors of 180-day mortality. Variables whose *p*-value was less than 0.1 in the univariable analysis or those that appeared to have significance that was useful clinically were incorporated in the multivariable adjustment. Findings were expressed in Hazard Ratios (HR) and 95% Confidence Intervals (CI).

The incremental prognostic value of including the SI trajectory in the baseline clinical model was determined by the Harrells Concordance Index (C-index), Net Reclassification Improvement (NRI) and Integrated Discrimination Improvement (IDI). Receiver Operating Characteristic (ROC) curves, Calibration plots and Decision Curve Analysis (DCA) were further used to visualize model performance. All the statistical testing was done with the help of R (Version 4.3.1) software with *p*-value less than 0.05 deemed to be statistically significant.

## 3 Results

### 3.1 Identification of dynamic Sarcopenia Index trajectories

**Figure 1:**
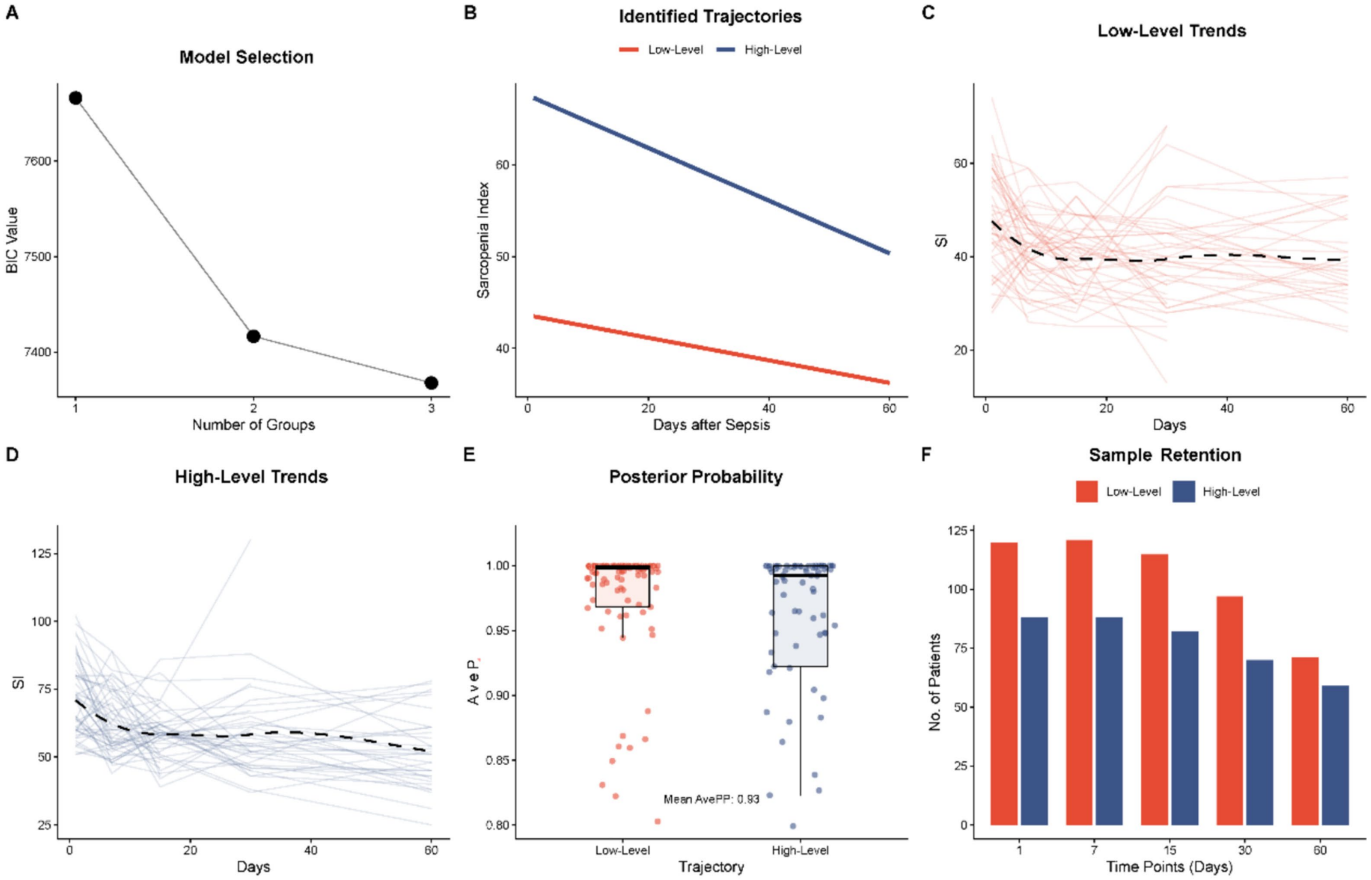
Identification and characteristics of Sarcopenia Index (SI) dynamic trajectories using Group-Based Trajectory Modeling (GBTM). **(A)** Model selection process based on the Bayesian information criterion (BIC), indicating the optimal number of groups. **(B)** The identified latent trajectory classes: the high-level group (blue line) and the Low-Level Group (red line) over the 60 day observation period. **(C,D)** Spaghetti plots showing individual SI measurements and the fitted mean trends for the High-Level and Low-Level Groups, respectively. **(E)** Distribution of average posterior probabilities (AvePP) for group assignment, demonstrating good model fit. **(F)** Bar chart illustrating sample retention and data availability at each time point (Day 1, 7, 15, 30, and 60) for both groups.

Group-Based Trajectory Modeling (GBTM) was employed to identify the patterns of latent longitudinal changes of the Sarcopenia Index (SI) after the emergence of sepsis. The Bayesian Information Criterion (BIC) was used as the principal model selection across models with low values that reflected a better fit-complexity relationship. As shown in Figure 1A, a significant decrease in BIC was observed between the one-group model (7665.85) and two-group model (7416.52). The three group model showed an additional slight decrease (7367.82), which was however still dropped in favor of a two group solution to have sufficient sample size in each stratum as well as to allow clinical interpretation.

Therefore, there were two different cubic trajectories found (Figure 1B). The majority of the cohort was represented in the first stage, the so-called Low-Level Group (*n* = 122). A significantly suppressed baseline SI of about 43.5 that was shown to have a consistent downward trend and a low of 36.2 after 60 days typified this group. The second group consisting of 88 individuals that was termed the High-Level Group (*n* = 88) on the other hand had a much retained mass of baseline muscle with an initial SI of 67.3. Although such group had a greater beginning, it also had a downwards trend, reducing to 50.3, during the 60-day survey period.

Model fit statistics substantiated the robustness of this classification. The mean posterior probabilities (AvePP) that group membership assignment was correct were greater than the 0.7 that is recommended (Figure 1E) which is high classification accuracy. Also, the spaghetti plots (Figures 1C,D) showed that the separate trajectories of patients versus the projected group means were very coherent. The longitudinal data was equally adequate to perform trajectory estimation during the follow-up even though there was anticipated loss as a result of either death or discharge (Figure 1F).

### 3.2 Baseline characteristics stratified by trajectories

**Figure 2:**
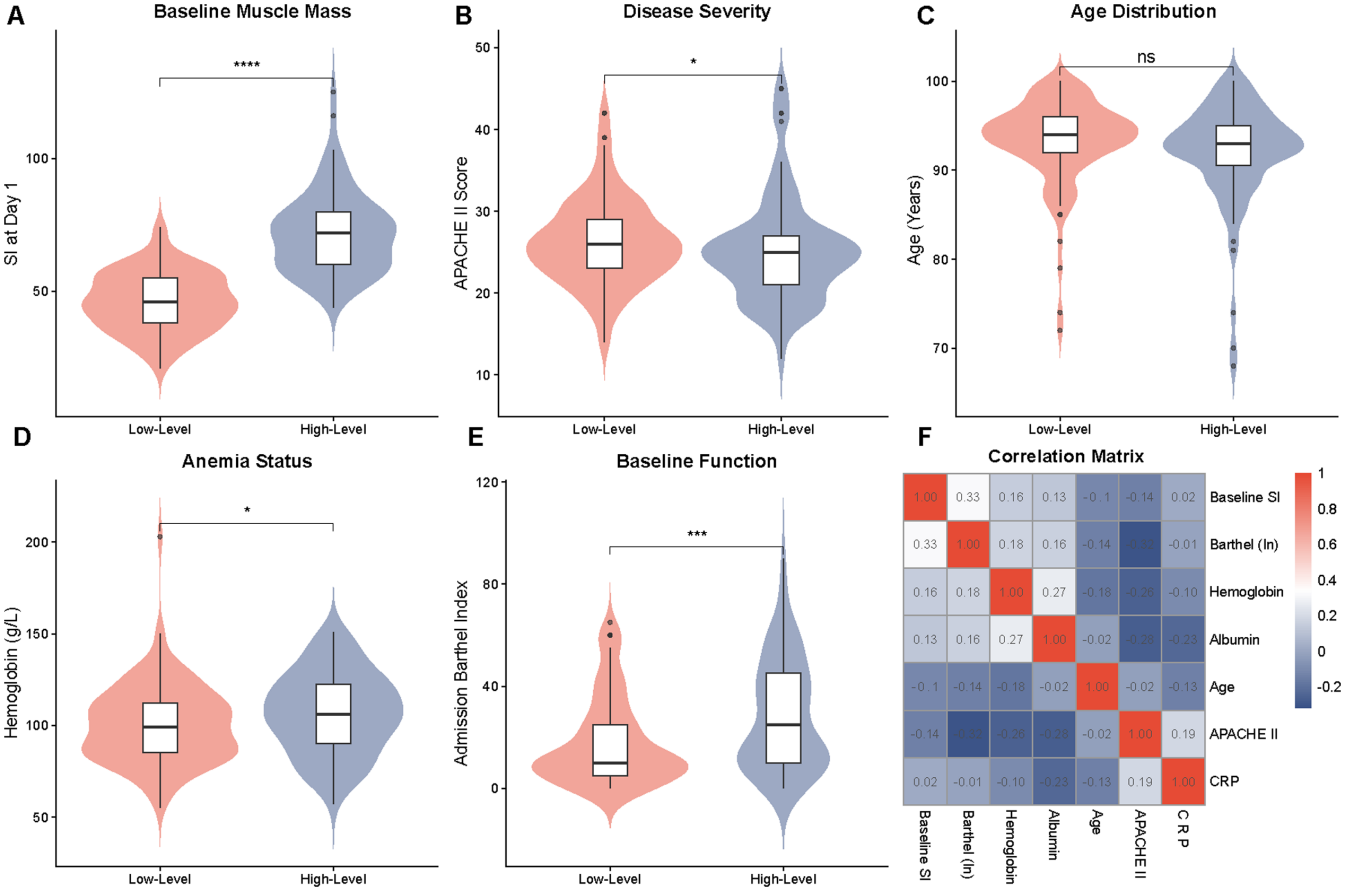
Comparison of baseline clinical characteristics and functional status between SI trajectory groups. **(A)** Violin plot comparing baseline muscle mass (SI at Day 1). **(B)** Comparison of disease severity (APACHE II score). **(C)** Comparison of age distribution. **(D)** Comparison of anemia status (hemoglobin levels). **(E)** Comparison of baseline function (Barthel Index). The Low-Level Group exhibited significantly lower baseline SI, hemoglobin, and Barthel Index scores compared to the High-Level Group. **(F)** Correlation matrix heatmap visualizing the relationships between SI, inflammatory markers, nutritional status, and clinical scores. **** indicates *p* < 0.0001, *** indicates *p* < 0.001, * indicates *p* < 0.05.

The comparison of the baseline characteristics between Low-Level Group (*n* = 122) and High-Level Group (*n* = 88) is provided in Table 1. The study population represented a super-elderly cohort with a mean age of 92.82 years. Demographic analysis indicated that there was no statistically significant heterogeneity in the two trajectories in terms of the age (*p* = 0.060; Figure 2C) or gender distribution (*p* = 0.091). The comorbidity burden was large, and equally divided, however no significant differences were found in the prevalence of hypertension (75.4% vs. 71.6%), coronary heart disease (59.8% vs. 60.2%), or active cancer (78.7% vs. 68.2%), indicating that the difference in SI trajectories was not mainly brought about by the predisposed chronic disease profile.

**Table 1:** Baseline characteristics of super-elderly sepsis patients stratified by Sarcopenia Index trajectories.

Variable	Total	High-level	Low-level	*p-*value
*n*	210	88	122	
Demographics				
Age, years	92.82 (5.14)	92.03 (5.68)	93.39 (4.65)	0.060
Male, *n* (%)	19 (9.0)	4 (4.5)	15 (12.3)	0.091
Comorbidities, *n* (%)				
Hypertension	155 (73.8)	63 (71.6)	92 (75.4)	0.644
Coronary heart disease	126 (60.0)	53 (60.2)	73 (59.8)	1.000
Diabetes mellitus	95 (45.2)	33 (37.5)	62 (50.8)	0.076
COPD	108 (51.4)	48 (54.5)	60 (49.2)	0.530
History of stroke	8 (3.8)	2 (2.3)	6 (4.9)	0.533
Active cancer	54 (25.7)	28 (31.8)	26 (21.3)	0.119
Long-term bedridden	151 (71.9)	48 (54.5)	103 (84.4)	<0.001
Sepsis characteristics				
COVID-19 infection, *n* (%)	22 (10.5)	9 (10.2)	13 (10.7)	1.000
APACHE II score	25.50 [22.00, 28.00]	25.00 [21.00, 27.00]	26.00 [23.00, 29.00]	0.034
SOFA score	8.00 [6.00, 11.00]	8.00 [6.00, 11.00]	8.50 [6.00, 11.00]	0.396
Functional status				
Barthel index (admission)	15.00 [10.00, 35.00]	25.00 [10.00, 45.00]	10.00 [5.00, 25.00]	<0.001
Δ Barthel (discharge − Adm)	−10.00 [−25.00, 0.00]	−10.00 [−30.00, 0.00]	−10.00 [−23.75, 0.00]	0.801
Laboratory findings				
Hemoglobin (g/L)	102.50 [87.00, 117.00]	106.00 [90.00, 122.25]	100.00 [85.00, 112.75]	0.030
Albumin (g/L)	32.25 [29.92, 35.40]	32.45 [30.38, 35.90]	31.90 [29.72, 34.88]	0.114
C-reactive protein (mg/L)	74.48 [41.87, 130.92]	74.12 [42.98, 131.07]	74.48 [37.96, 129.01]	0.617
Procalcitonin (ng/mL)	1.30 [0.50, 4.02]	1.19 [0.48, 4.95]	1.36 [0.50, 3.65]	0.693
D-dimer (mg/L)	1.88 [1.12, 4.23]	1.89 [0.98, 4.58]	1.87 [1.15, 3.71]	0.918
Median baseline SI (Day 1)	56.00 [44.00, 70.75]	72.00 [60.00, 80.00]	46.00 [38.00, 55.75]	<0.001
Outcomes				
180-day mortality, *n* (%)	119 (56.7)	43 (48.9)	76 (62.3)	0.072

Values are presented as mean ± SD for normally distributed variables, median [IQR] for non-normally distributed variables, or *n* (%) for categorical variables.

*p*-values were calculated using Student’s *t*-test, Mann–Whitney *U* test, or Chi-square test as appropriate.

COPD, Chronic Obstructive Pulmonary Disease; APACHE II, Acute Physiology and Chronic Health Evaluation II; SOFA, Sequential Organ Failure Assessment.

Nevertheless, there was a sharp contrast in physiological reserves and functional status. As measured by Table 1 and presented in Figure 2E, the Low-Level Group demonstrated severe functional impairment, with a median Barthel Index of 10.0 (IQR: 5.00–25.00) compared to 25.0 (IQR: 10.00–45.00) in High-Level Group (*p* < 0.001). This functional impairment matched with significantly larger proportion of long-term bedridden in the Low-Level Group (84.4% vs. 54.5%, *p* < 0.001) with a somewhat higher APACHE II score (median 26.00 vs. 25.00, *p* = 0.034; Figure 2B), suggesting an increased general physiological dysfunction. Intergroup differences in laboratory findings were specific rather than systemic. There were no significant differences in the inflammatory markers, such as C-reactive protein (*p* = 0.617), procalcitonin (*p* = 0.693), and D-dimer (*p* = 0.918). On the other hand, hemoglobin concentrations were substantially lower in the Low-Level Group (median 100.00 vs. 106.00 g/L, *p* = 0.030; Figure 2D) indicating that there is in fact a specific relation between muscle depletion and anemia.

In line with the trajectory classification, the absolute median of Baseline Sarcopenia Index at Day 1 in Low-Level Group was significantly decreasing compared to the High-Level Group (46.00 vs. 72.00, *p* < 0.001, Figure 2A). These results were further corroborated by the correlation matrix heatmap (Figure 2F), which found that the baseline SI was positively correlated with functional markers (Barthel Index) and nutritional status (hemoglobin) and negatively correlated with age and APACHE II scores, indicating the existence of a multifaceted frailty-anemia-disease complex of frailty potentially contributing to the high-risk group.

### 3.3 Association between SI trajectories and 180-day mortality

**Figure 3:**
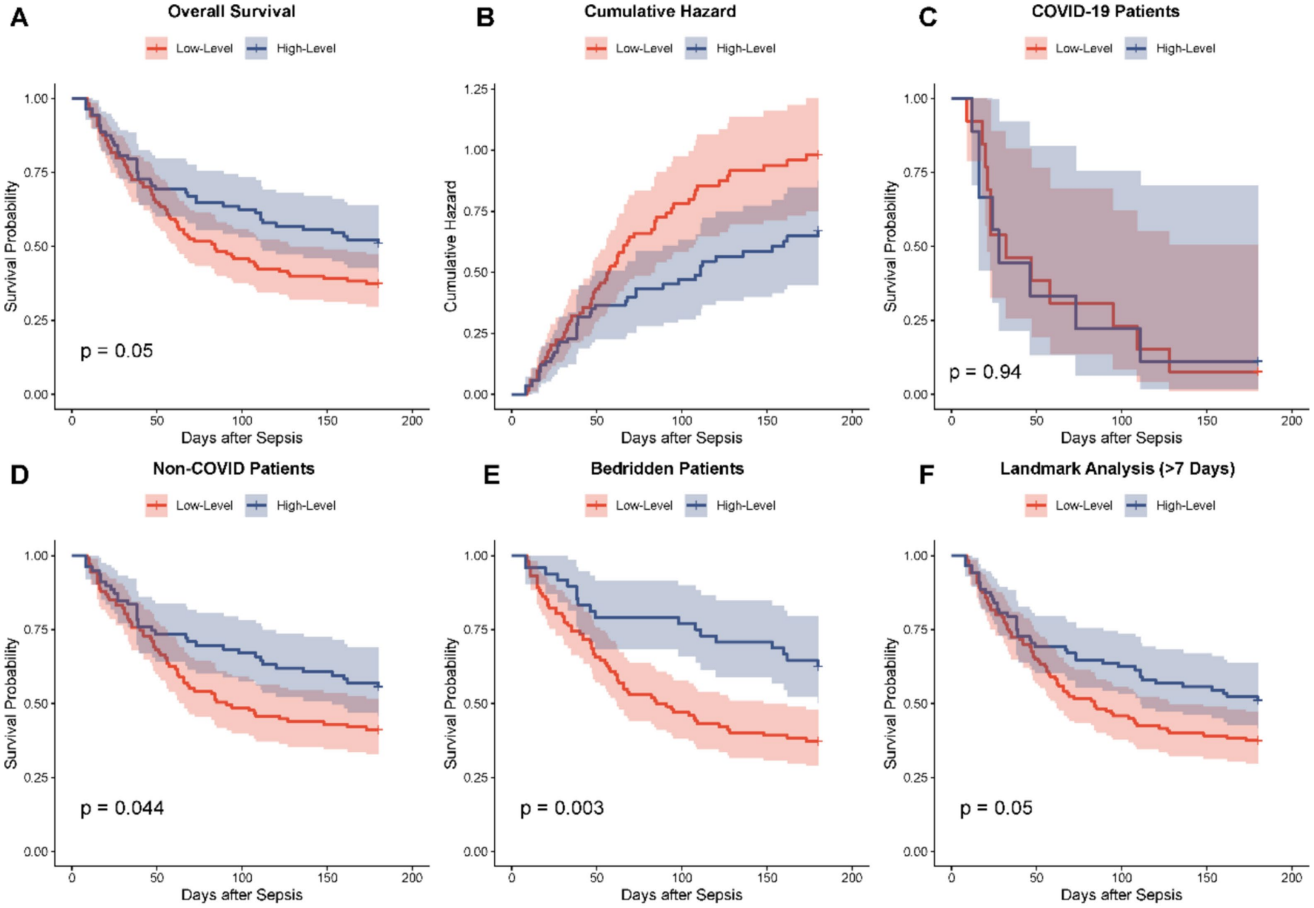
Kaplan–Meier survival analysis and cumulative hazard curves for 180-day mortality stratified by SI trajectory groups. **(A)** Overall Kaplan–Meier survival curves showing significantly lower survival probability in the Low-Level Group compared to the High-Level Group (*p* = 0.05). **(B)** Cumulative hazard curves for 180-day mortality. **(C–F)** Stratified survival analyses: **(C)** Patients with COVID-19 (*p* = 0.94); **(D)** Patients without COVID-19 (*p* = 0.044); **(E)** Long-term bedridden patients (*p* = 0.003), showing a marked divergence in survival; **(F)** Landmark analysis excluding early deaths within 7 days (*p* = 0.05).

Building upon the observed baseline disparities, Kaplan–Meier survival analysis was conducted to delineate the prognostic implications of the SI trajectories over the 180-day follow-up period. Figure 3A below shows that the total survival rate line in the two groups differed, with the Low-Level Group having a steadily sharp rate of decrease than that of the High-Level Group (Log-rank *p* = 0.05). This survival disadvantage was also supported by the cumulative hazard curves (Figure 3B) which were showing an increased accrual of a mortality risk in the Low-Level trajectory over time. Stratified analyses showed that although the survival difference was not significant among patients with COVID-19 (Figure 3C, *p* = 0.94), this prognostic separation was especially prominent in those uninfected with COVID-19 (Figure 3D, *p* = 0.044) and in the bedridden population (Figure 3E, *p* = 0.003) clinical subgroups, implying that the prognostic role of the trajectory is maintained in patients within particular high-risk clinical subgroups. Furthermore, a landmark analysis excluding early deaths within 7 days confirmed the robustness of this long-term survival disadvantage (Figure 3F, *p* = 0.05).

Cox proportional hazards regression analyses were conducted to adjust a possible presence of confounders that have been observed in the baseline characteristics, e.g., age, disease severity (APACHE II) and comorbidities (Table 2). The Low-Level trajectory had a borderline relationship with the risk of increased mortality (Hazard Ratio [HR] = 1.44, 95% CI: 0.99210, *p* = 0.056) in the univariate analysis. Nonetheless, the association was statistically significant after the correction of the age, gender, APACHE II score, active cancer and COVID-19 infection as well as the bedridden status in the multivariate model. Assignment to the Low-Level Group was identified as an independent predictor of 180-day mortality, associated with a 64% increase in the hazard of death (Adjusted HR = 1.64, 95% CI: 1.08–2.48, *p* = 0.020).

**Table 2:** Univariate and multivariate cox proportional hazards analysis for 180-day mortality.

Variable	Univariate HR (95% CI)	*p*-value	Multivariate HR (95% CI)	*p-*value
Age (per year)	1.06 (1.01–1.10)	0.010	1.06 (1.01–1.10)	0.008
Gender (male vs. female)	0.78 (0.43–1.42)	0.424	0.51 (0.27–0.97)	0.039
APACHE II score	1.04 (1.01–1.07)	0.008	1.05 (1.02–1.08)	0.003
Active cancer (Yes vs. No)	2.16 (1.48–3.15)	<0.001	2.45 (1.66–3.64)	<0.001
COVID-19 (Yes vs. No)	2.93 (1.80–4.77)	<0.001	3.15 (1.90–5.21)	<0.001
Long-term bedridden (Yes vs. No)	0.78 (0.53–1.16)	0.220	0.58 (0.37–0.91)	0.017
SI trajectory (low-level)	1.44 (0.99–2.10)	0.056	1.64 (1.08–2.48)	0.020

^a^Multivariate analysis was adjusted for age, gender, APACHE II, active cancer, COVID-19, and bedridden status.

HR, hazard ratio; CI, confidence interval.

Also, the multivariable model proved the relevance of the important influence of acute and chronic comorbidities on prognosis. Active cancer (HR = 2.45, 95% CI: 1.66364) and COVID-19 infection (HR = 3.15, 95% CI: 1.905.21) continued to be strong predictive variables of death indicating the complicated clinical context of this super-elderly group.

### 3.4 Incremental predictive value of adding SI trajectory

**Figure 4:**
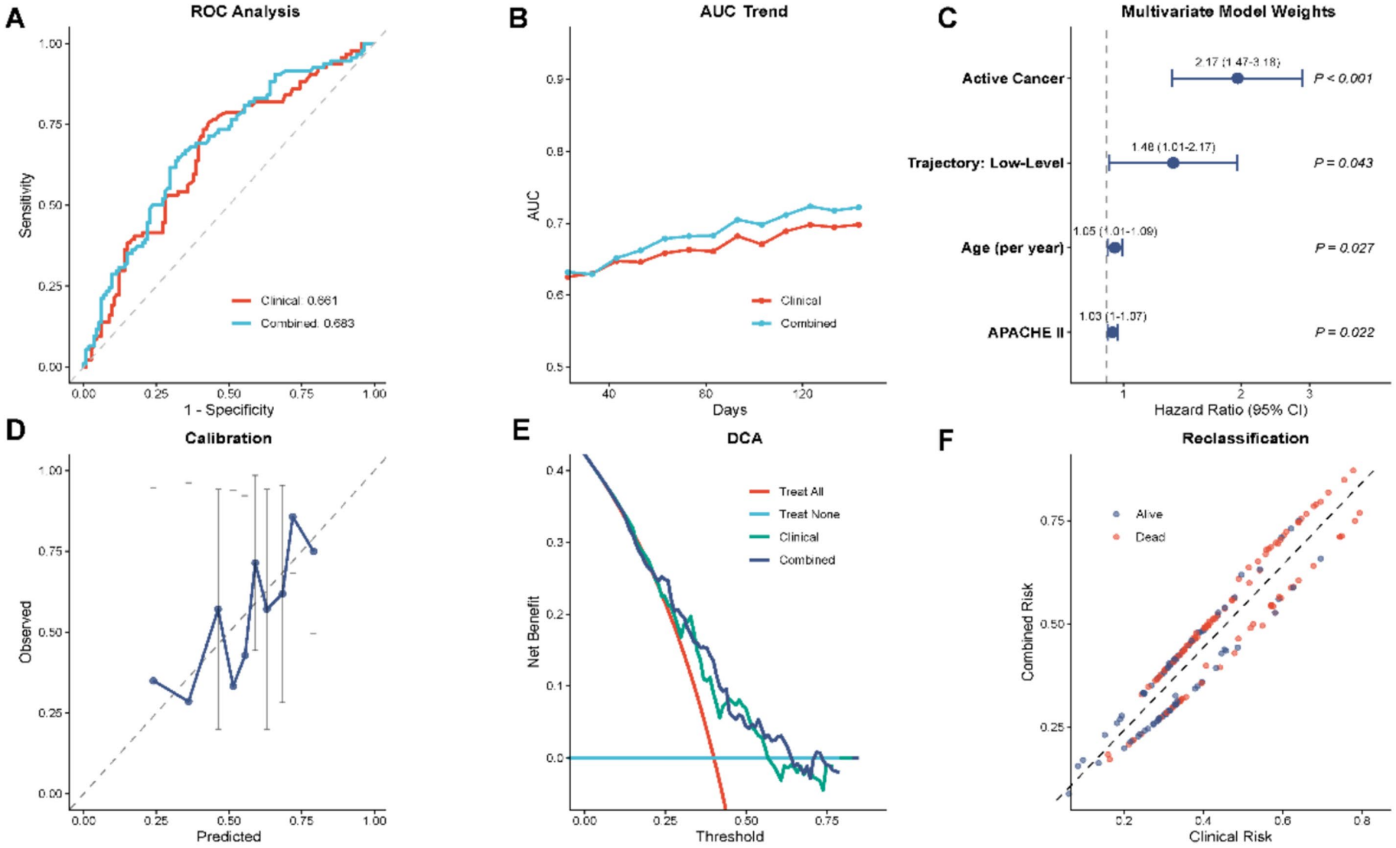
Predictive performance evaluation and multivariable Cox regression analysis. **(A)** Receiver operating characteristic (ROC) curves comparing the discrimination ability of the clinical model versus the combined model (clinical + SI trajectory). **(B)** Time-dependent AUC trend demonstrating the stability of predictive performance over time. **(C)** Forest plot representing the multivariable Cox proportional hazards model; variables include active cancer, SI trajectory (low-level), age, and APACHE II score. **(D)** Calibration curve showing the agreement between predicted and observed probabilities. **(E)** Decision curve analysis (DCA) comparing the net benefit of different models. **(F)** Reclassification plot illustrating risk stratification improvement.

To ascertain whether the dynamic SI trajectory offers prognostic utility beyond standard clinical parameters, predictive performance was compared between a baseline clinical model (Model 1) and a trajectory-enhanced model (Model 3). The basic model of age, APACHE II score, and active cancer status showed a concordance index (C-index) of 0.631 (95% CI, 0.5790684): as shown in Table 3. Introduction of SI trajectory into this framework (Model 3) also led to an increase in the C-index after introduction to 0.643 (95 percent interval, 0.592 to 0.694). Although the change in discrimination was comparatively slight, changes in classification of risks are statistically significant. The Net Reclassification Improvement (NRI) was estimated as 0.020 (*p* < 0.05) and Integrated Discrimination Improvement (IDI) was estimated as 0.150 (*p* = 0.078). These metrics suggest that while trajectory information significantly improved classification accuracy (NRI), the improvement in integrated discrimination did not reach statistical significance. Figure 4 visually supports these statistical results by the model evaluation measures. Figure 4A shows that the Receiver Operating Characteristic (ROC) curves of the Combined Model (red line), or Clinical Model (blue line), always have a larger area under the curve than the other. Moreover, as indicated in the Decision Curve Analysis in Figure 4E, the trajectory-enhanced model has a better net benefit in a large set of threshold probabilities, which implies more clinical use. Figure 4D that shows the calibration curve showed good consistency in predicted values of the probability and observed values, showing that the estimates done by the combined model were satisfactory. Taken together, these metrics imply that the SI trajectory provides non-interactive and cumulative prognostic tasks, which help to identify high-risk super-elderly patients.

**Table 3:** Incremental predictive value of adding SI trajectory to clinical risk models (at 180 days).

Model	C_Index (95% CI)	NRI (95% CI)	*p*-value	IDI (95% CI)	*p*-value
Model 1	0.631 (0.579–0.684)	—	—	—	—
Model 2 (SI)	0.638 (0.586–0.690)	0.009 (−0.001, 0.037)	0.235	0.135 (−0.070, 0.250)	0.235
Model 3 (Traj)	0.643 (0.592–0.694)	0.020 (0.000, 0.056)	<0.05	0.150 (−0.008, 0.271)	0.078

### 3.5 Subgroup analysis and sensitivity analysis

**Figure 5:**
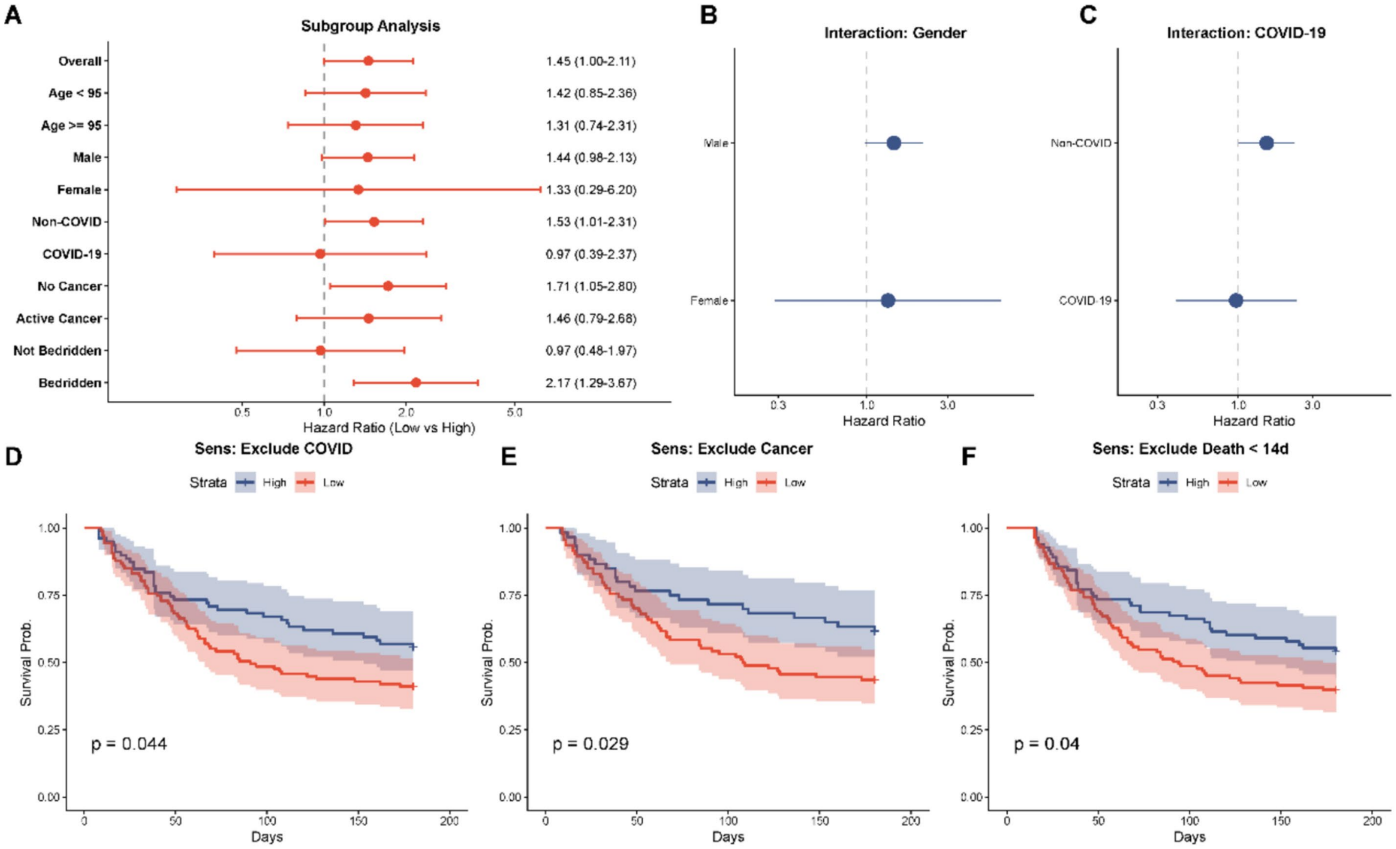
Subgroup analyses and sensitivity analyses assessing the robustness of the association between low-level SI trajectory and mortality. **(A)** Forest plot of subgroup analyses stratified by age, gender, COVID-19 status, active cancer, and bedridden status. The hazard ratios (HR) consistently favor the high-level group across most strata. **(B,C)** Interaction plots for gender and COVID-19 status. **(D–F)** Kaplan–Meier sensitivity analyses verifying the consistency of findings after: **(D)** excluding COVID-19 patients; **(E)** excluding patients with active cancer; and **(F)** excluding deaths within 14 days.

To evaluate the consistency of the association between the Low-Level SI trajectory and 180-day mortality across different clinical strata, subgroup analyses were performed as depicted in the forest plot (Figure 5A). The Low-Level Group hazard ratios (HRs) tended to increase the risk of mortality (HR > 1.00) in most of the predetermined subgroups thus indicating that the prognostic value of the SI trajectory is usually strong.

It is important to mention that it was the predictive power of the trajectory that was stronger in some high-risk populations. Among patients with history of long-term bedridden, the risk of death among the Low-Level Group was more than two times (HR = 2.17, 95% CI: 1.293.67, *p* = 0.004). They were also significantly associated in patients without active cancer (HR = 1.71, 95% CI: 1.052.80, *p* = 0.031) and among patients that were not infected with COVID-19 (HR = 1.53, 95% CI: 1.012.31, *p* = 0.046).

On the other hand, statistical significance were not achieved in the non-bedridden, female and COVID-19 positive subsets. As Figure 5B shows, the direction of risk was similar between gender (Male HR 1.44 vs. Female HR 1.33) but the super-large confidence intervals of the female subgroup would indicate the relatively small sample size (*n* = 18). On the same note, in the COVID-19 subgroup (Figure 5C) the association was less significant (HR = 0.97, *p* = 0.942) and this is possibly due to small size of the subgroup (*n* = 22) or the acute effect of the viral infection itself concealing the risks of sarcopenia in the baseline.

The Kaplan–Meier curve-based sensitivity analysis (Figures 5D–F) was another test that confirmed the validity of the findings. The survival disadvantage of the Low-Level Group remained even post-removal of patients with COVID-19 and active cancer in addition to early deaths occurring within 14 days, which confirmed that the main findings were not pushed by these particular confounders or acute events of early mortality.

## 4 Discussion

The present study successfully applied Group-Based Trajectory Modeling (GBTM) to elucidate the longitudinal evolution of the Sarcopenia Index (SI) in a super-elderly cohort with sepsis. Two different dynamic phenotypes were distinguished: a ‘High-Level Group’ with relatively preserved initial muscle mass and a ‘Low-Level Group’ characterized by profound baseline depletion and a persistent declining trend. A primary finding of this investigation is that assignment to the Low-Level trajectory constitutes a potent and independent predictor of 180-day mortality, even after adjusting for established risk variables, including age, APACHE II score, and active malignancy, have been controlled. Furthermore, the integration of SI trajectory data into a conventional clinical risk model yielded modest improvements in risk stratification, as evidenced by a significant NRI, although the IDI showed only a borderline trend. This highlights the incremental prognostic value of dynamic muscle mass monitoring over static baseline assessments in this vulnerable population.

The results of our study are consistent with other sources that embrace the idea of the so-called sepsis-sarcopenia vicious cycle, where inflammation promotes muscle loss, and muscle loss further impairs metabolic reserve (15). Along with the systemic inflammation being a symptom of sepsis, it is noteworthy that, in our cohort, the inflammatory markers of CRP and procalcitonin did not significantly differ between the two trajectory groups (Table 1). Rather, the divergence was fuelled by the functional status (Barthel Index) and nutritional reserves (Hemoglobin). This implies that prognosis in this super-elderly cohort may be determined less by the magnitude of the acute inflammatory surge and more by the patient’s baseline physiological reserve, a concept closely aligned with the frailty phenotype (16). The Low-Level trajectory is probably due to a critically low, that is, a very low reserve, in which the catabolic stress of sepsis overwhelms the recovery ability of the body. Interestingly, there is a decreasing pattern in SI as shown in Figure 1B even among the High-Level Group. This global decrease confirms the earlier research that sepsis causes a hypercatabolic condition with severe skeletal muscle atrophies caused by ubiquitin-proteasome pathway and cytokine storm (17–19). This universal decline suggests that no patient is entirely spared from sepsis-induced muscle depletion (20). However, a higher baseline muscle mass, as seen in the High-Level Group, still appears to confer a significant survival advantage (21). Our findings align with imaging-based studies, such as those by Looijaard et al. (22), who reported that low skeletal muscle area on CT is a strong predictor of ICU mortality. While CT remains the gold standard, our study demonstrates that the SI trajectory offers a comparable prognostic signal. Quantitatively, the hazard ratios observed in our Low-Level Group (HR 1.64) are consistent with the increased mortality risk reported in CT-defined sarcopenia cohorts (HR range 1.5–2.2). Notably, our multivariate model suggested a protective effect of long-term bedridden status (HR 0.58). This seemingly paradoxical finding may reflect a selection bias where only the physiologically robust bedridden patients survived the initial septic insult to be hospitalized. Alternatively, once severity (APACHE II) and muscle mass (SI) are controlled for, bedridden patients may have lower acute metabolic expenditures compared to previously active patients undergoing sudden catabolic crash.

In the context of subgroup analyses (Figure 5), the relationships between the trajectory of Low-Level corresponding with mortality were still significant in the majority of clinical strata, especially the long-term bedridden patients. But statistical significant was diluted in the female and the COVID-19 group. The wide confidence intervals observed in these subgroups are likely attributable to the limited sample size (e.g., 18 females and 22 COVID-19 patients) rather than a true biological lack of association (23). Although statistical significance was not reached, the point estimates of the hazard ratios in these subgroups still trended towards an increased risk for the Low-Level Group (Figures 5B,C), suggesting the limitation may stem from insufficient statistical power rather than a true effect modification (24). Further research on bigger sample sizes should be conducted to authenticate these particular interactions.

There are a number of limitations of the study that should be noted. To begin with, the study is retrospective and single center and this may restrict the extrapolation of the results to a different population or health care environment. Second, the SI is a validated surrogate measure of muscle mass though we did not have direct gold standard measures (which could be CT or MRI-based quantification of muscle area) in such a cohort. Third, GBTM provides a complex way of dealing with longitudinal data, the classification is probabilistic rather than deterministic, and the modest sample size (*n* = 210) may have limited the power to identify more subtle trajectory subgroups. Furthermore, the requirement for at least three SI measurements potentially introduces survivor bias, as patients who died rapidly after admission were excluded. This may result in an underestimation of the association between muscle depletion and mortality in the most fulminant cases. Lastly, the confounding factors (measured or not) may have contributed to the changes in SI, which include pre-admission dietary consumption. Furthermore, relying solely on SI oversimplifies the complex biology of aging. Future research should aim to integrate SI trajectories with inflammatory panels (e.g., IL-6) and comprehensive geriatric assessments to create a multidimensional prognostic model that better captures the heterogeneity of the super-elderly. In summary, our findings indicate that a low and declining Sarcopenia Index trajectory is strongly associated with poor prognosis in super-elderly patients with sepsis. The SI trajectory is a useful and easily available biomarker that simultaneously reflects the dynamic relationship between frailty and acute disease and provides a new risk stratification option and could be the basis of future nutritional or rehabilitative interventions. From a broader perspective, our findings highlight the critical role of “physiological reserve” in determining survival against sepsis insults. The GBTM approach used here to map muscle loss could similarly be applied to other dynamic organ functions in intensive care, such as renal recovery trajectories or cognitive decline patterns. Understanding these dynamic phenotypes allows for a shift from static risk assessment to personalized, time-variant physiological monitoring in critical care medicine.

## 5 Conclusion

This study successfully identified two distinct dynamic trajectories of the Sarcopenia Index (SI) of super-elderly patients with sepsis in the presence of Group-Based Trajectory Modeling. The Low-Level trajectory, characterized by a steep profile depicting deep and sequential muscle loss was found to be a significant and autonomous predictor of 180-day mortality. In addition, combination of SI trajectory information significantly enhanced the predictive ability of known clinical risk models as shown by reclassification measure (NRI and IDI). These results are indicative that patients in geriatric acute care have dynamic SI monitoring as a feasible, available surrogate that measures physiological reserve. Timely detection of patients along this high-risk path might help to further classify the risks and implement specific nutrition and rehabilitation options that will help to reduce the negative prognostic influence of sepsis-related sarcopenia.

## Data Availability

The raw data supporting the conclusions of this article will be made available by the authors, without undue reservation.
